# Functional Analysis of an Acyltransferase-Like Domain from Polyunsaturated Fatty Acid Synthase in Thraustochytrium

**DOI:** 10.3390/microorganisms9030626

**Published:** 2021-03-17

**Authors:** Carla Almendáriz-Palacios, Dauenpen Meesapyodsuk, Xiao Qiu

**Affiliations:** 1 Department of Food and Bioproduct Sciences, University of Saskatchewan, Saskatoon, SK S7N 58A, Canada; c.almendariz@usask.ca; 2 National Research Council of Canada, Saskatoon, SK S7N 0W9, Canada; dauenpen.meesapyodsuk@nrc-cnrc.gc.ca

**Keywords:** PUFA synthase, acyltransferase-like domain, DHA, Thraustochytrium

## Abstract

Biosynthesis of very long chain polyunsaturated fatty acids (VLCPUFA) such as docosahexaenoic acid (DHA, 22:6-4,7,10,13,16,19) and docosapentaenoic acid (DPA, 22:5-4,7,10,13,16) in protist Thraustochytrium is catalyzed by a polyunsaturated fatty acids (PUFA) synthase comprising three large subunits, each with multiple catalytic domains. This study used complementation test, in vitro assays, and functional expression to characterize an acyltransferase (AT)-like domain in Subunit-B of a PUFA synthase from Thraustochytrium. Complementation test in *Escherichia coli* showed that the AT-like domain could not restore the growth phenotype of a temperature-sensitive mutant (*∆fabD^ts^*) defective in malonyl-CoA:ACP transacylase activity. In vitro assays showed that the AT-like domain possessed thioesterase activity towards a few acyl-CoAs tested where docosahexaenoyl-CoA (DHA-CoA) was the preferred substrate. Expression of this domain in an *E. coli* mutant (*∆fadD*) defective in acyl-CoA synthetase activity resulted in the increased accumulation of free fatty acids. Site-directed mutagenesis showed that the substitution of two putative active site residues, serine at 96 (S96) and histidine at 220 (H220), in the AT-like domain significantly reduced its activity towards DHA-CoA and accumulation of free fatty acids in the *∆fadD* mutant. These results indicate that the AT-like domain of the PUFA synthase does not function as a malonyl-CoA:ACP transacylase, rather it functions as a thioesterase. It might catalyze the last step of the VLCPUFA biosynthesis by releasing freshly synthesized VLCPUFAs attached to ACP domains of the PUFA synthase in Thraustochytrium.

## 1. Introduction

Very long chain polyunsaturated fatty acids (VLCPUFAs) such as docosahexaenoic acid (DHA, 22:6n-3) and eicosapentaenoic acid (EPA, 20:5n-3) are essential components of cell membranes and precursors for eicosanoids and docosanoids involved in mediating various physiological processes in humans and animals [1]. Lack or deficiency of these fatty acids can have serious consequences such as abnormal growth and development, underperformance of vision and cognition, and various diseases. Therefore, humans and animals are encouraged to supplement these fatty acids in diets for better health, as they cannot be de novo synthesized [2,3]. The current sources of VLCPUFAs for humans and animals are oils from marine fish and oleaginous VLCPUFA-producing microorganisms. However, oil from oceanic fish has been over-exploited and oil from the microbes is expensive due to the high cost in microbial culture and oil extraction. Metabolic engineering of oilseed plants using the biosynthetic genes from VLCPUFA-producing microorganisms has thus been considered as a potential alternative way to supply these fatty acids [4,5].

Two distinct biosynthetic pathways of VLCPUFAs exist in nature [6]. The aerobic pathway uses alternating desaturations and elongations to synthesize VLCPUFAs where double bonds are introduced by desaturases, and chain lengths are determined by elongases. This biosynthetic pathway occurs mainly in higher animals and eukaryotic microorganisms and requires molecular oxygen as a co-factor for desaturations [7,8]. The anaerobic pathway utilizes a single polyunsaturated fatty acid (PUFA) synthase to synthesize VLCPUFAs [9]. Similar to polyketide synthase (PKS), PUFA synthase is a mega-enzyme comprising multiple catalytic domains such as ketoacyl synthase (KS), malonyl-CoA:ACP acyltransferase (MAT), dehydratase (DH), ketoacyl reductase (KR), enoylreductase (ER), and acyl carrier protein (ACP) [10]. This pathway takes place only in marine microorganisms (both eukaryotic and prokaryotic) and does not require oxygen-dependent desaturations to introduce double bonds. Instead, double bonds are introduced during the acyl chain extension process, as seen in the biosynthesis of unsaturated fatty acids in *Escherichia coli* (*E. coli*) [11]. Biosynthesis of VLCPUFAs by a PUFA synthase, like biosynthesis of long chain fatty acids by Type I and Type II fatty acid synthases (FAS), proceeds with reiterative cycles using malonate as a two-carbon donor and acyl carrier protein (ACP) as the covalent attachment for acyl chain extension. A full cycle also comprises four reactions: condensation, ketoacyl reduction, dehydration, and enoyl reduction. However, unlike long chain fatty acid synthesis, VLCPUFA synthesis catalyzed by a PUFA synthase can periodically skip the last enoyl reduction step of a full cycle, resulting in a double bond being introduced in an acyl chain [12].

*Thraustochytrium* sp. 26185 is a unicellular marine protist that produces a high level of DHA. Our recent genome sequencing indicated both aerobic pathway and anaerobic pathway for the biosynthesis of DHA co-existed in Thraustochytrium [13]. However, our in vivo feeding and in vitro assays showed that the aerobic pathway was not functional, and the anaerobic pathway was solely responsible for the biosynthesis of DHA in the protist [14,15]. The PUFA synthase from Thraustochytrium comprises three large subunits, each with multiple catalytic domains. All these domains are predicted on the presence of characteristic catalytic motifs in the primary sequence, and the exact functions of most of them remain to be determined except for ketoacyl synthase and dehydratase domains of PUFA synthases [16,17,18,19,20]. The purpose of this study was to functionally analyze an acyltransferase (AT)-like domain from Subunit-B of a PUFA synthase in Thraustochytrium through complementation test, site-directed mutagenesis and in vitro assays in *E. coli*. The results provide evidence that this domain functions as a thioesterase probably involved in releasing freshly synthesized VLCPUFAs as free fatty acids from their thioesters attached to the PUFA synthase in Thraustochytrium.

## 2. Materials and Methods

### 2.1. Strains, Plasmids, Kits, and Primers

*E. coli* Top10 was purchased from Invitrogen Biotechnology Co. (Grand Island, NY, USA). *E. coli fabD* mutant (*ΔfabD*) was obtained from the Coli Genetic Stock Center from the Yale University (New Haven, CO, USA). *E. coli ∆fadD* strain was provided by Dr. Pamela Silver, Harvard Medical School (Boston, MA, USA) [21]. *E. coli* SHuffle strain was acquired from New England BioLabs (Ipswich, MA, USA) (Table S1). The pCDFDuet-1 vector with the ORF-B of the PUFA synthase from *Thraustochytrium* sp. 26185 was obtained from our previous research [14]. Expression vectors, pBAD and pET28a, were purchased from Invitrogen Co. (Carlsbad, CA, USA) (Table S2). DNA purification kit was acquired from Bio Basic Inc. (York, ON, Canada). Q5 polymerase, restriction enzymes and dNTP were purchased from New England Biolabs (NEB) (Ipswich, MA, USA). HP Taq DNA polymerase was purchased from Bio Basic Inc. T4 ligase was acquired from Thermo Fisher Scientific. EcoRI, HindIII, and BglII restriction enzymes were purchased from NEB. Primers for MAT PCR amplifications and site-directed mutagenesis (Table S3) were synthesized by Sigma-Aldrich (St. Louis, MO, USA).

### 2.2. Sequence Analysis of the PUFA Synthase from Thraustochytrium

Polyunsaturated fatty acid synthase from *Thraustochytrium* sp. ATCC 26185 (GenBank accession no. AOG21005.1) in the National Center for Biotechnology Information website (NCBI) was analyzed by BLASTp (NCBI), MegAlign (DNASTAR), and SWISS-MODEL. The boundaries of the AT-like domain were determined by sequence alignment and homology modeling. Amino acid sequence of the Subunit-B of the PUFA synthase was used as a query to search NCBI protein database by BLASTp with the default algorithm parameters to identify the AT-like domain and its homologous sequences. Three homologous MAT proteins from *Streptococcus pneumoniae*, *Coxiella burnetti*, and *E. coli* were used as templates for homology modelling. Conserved motifs and residues were identified by sequence alignment using the ClustalW method with the slow-accurate option to obtain a precise alignment even if the sequences were highly divergent.

### 2.3. Cloning and Expression of AT-Like Domain in E. coli

Two primers, AT-like (ORF-B) F and AT-like (ORF-B) R, were used to amplify the AT-like domain from ORF-B. Primers MAT (ORF-A) F and MAT (ORF-A) R were used to amplify the MAT domain from ORF-A. *E. coli* MAT was amplified using primers FabD-F and FabD-R (Table S3). For complementation assays, the amplified fragments were sub-cloned into pBAD vector digested by EcoRI and HindIII. For in vitro assay, the fragments were cloned into pET28a using the same restriction sites. The recombinant vectors were transformed into *E. coli* Top10 for propagation.

### 2.4. Complementation of E. coli FabD Mutant

A thermolabile malonyl-CoA:ACP transacylase (MAT) mutant (*ΔfabD*) defective in fatty acid synthesis was used for the complementation assay [22]. The strain could grow only at 37 °C (permissive temperature) but not at 42 °C (non-permissive temperature) [23]. This conditional mutant was used for the test because the complete genetic inactivation of the *MAT* gene was lethal in *E. coli* [24]. Besides pBAD_AT-like expressing the AT-like domain from Subunit-B, two positive controls, pBAD_FabD expressing wild type *E. coli* FabD and pBAD_MAT expressing the MAT domain from Subunit-A of the PUFA synthase, were also included in complementation assays. A single colony of each transformant was first grown in an ampicillin LB liquid medium at 37 °C until an OD_600_ = 0.4 was reached. After that, 2 μL of each culture were dotted on ampicillin LB plates containing 0.01% (*w/v*) of l-arabinose [25,26]. The duplicate plates were cultured overnight at 37 °C and 42 °C, respectively.

### 2.5. Expression of the AT-Like Domain in E. coli fadD Mutant

An *E. coli ∆fadD* strain was used to over-express the AT-like domain using pET28a_AT-like plasmid. The mutant with the empty vector was used as a negative control. The transformants were selected on kanamycin LB plates. A single colony from the plate was inoculated to 5 mL of kanamycin LB liquid medium and incubated overnight at 37 °C. An aliquot of 300 μL of the overnight culture was inoculated into 30 mL of LB medium and incubated at 37 °C. After an OD_600_ of the culture reached 0.5, isopropyl β- d-1-thiogalactopyranoside (IPTG) at the final concentration of 1 mM was used to induce the expression at 16 °C [26]. The cell pellet of 200 μL culture was resuspended in 20 μL of SDS-PAGE loading buffer (60 mM Tris-HCl pH 6.8, 2% SDS, 0.1% bromophenol blue, 25% glycerine, 14.4 mM β-mercaptoethanol) and analyzed for expressed proteins by electrophoresis [16].

### 2.6. Free Fatty Acid Analysis

Ten milliliters of the culture after 24 h of induction were centrifuged at 10,000 rpm for 10 min for collecting cell pellets and supernatant. Total lipids from cell pellets were extracted following the protocol of Folch and colleagues [27]. One milliliter of hot isopropanol was added to cell pellet and incubated at 80 °C for 10 min to inactivate enzymes. After the sample was cooled down to room temperature, total lipids from cells were extracted with 2 mL of chloroform and 1 mL of methanol. The mixture was centrifuged at 2200 rpm for 10 min, and the lower phase was transferred to a clean tube. Two milliliters of chloroform were added again to extract the residual lipids left in the sample. The lower phase was combined with the previous one. After that, 1 mL of KCl 0.88% (*w/v*) was added to extract any contaminated hydrophilic compounds in the lipid extract. The mixture was centrifuged at 2200 rpm for 10 min, and the aqueous phase was removed as much as possible. The organic solvent phase of extractions was dried under N_2_ gas [25]. Free fatty acids were selectively methylated to fatty acid methyl esters (FAMEs) using 20 μL of diazomethane (1:8, diazomethane:hexane) and 10 μL of methanol at room temperature for 5 min [28]. After the sample was dried under N_2_ gas, total FAMEs were dissolved in 50 μL of hexane and analyzed by gas-chromatography [29].

Extracellular free fatty acids extracted from the culture were done by mixing 2 mL of the supernatant with the same volume of hexane and 500 μL of NaCl saturated water (26.47%, *w/w*). The organic solvent phase of extractions was dried under N_2_ gas. Free fatty acids were selectively methylated as described above for cell pellets. After the sample was dried under N_2_ gas, total FAMEs were dissolved in 50 μL of hexane and analyzed by gas-chromatography. Ten microliters of 0.1 mg/L of heptadecanoic (17:0) were used as an internal standard. All analyses were performed in triplicate.

### 2.7. Expression of AT-Like Domain in E. coli SHuffle

*E. coli* SHuffle strain was used to express pET28a_AT-like for in vitro assays. A single colony of transformants was inoculated to 5 mL of kanamycin LB liquid medium and incubated overnight at 37 °C. An aliquot of 200 μL of the overnight culture was then inoculated into 20 mL and incubated at 30 °C until reaching an OD_600_ = 0.5. Subsequently, IPTG was used to induce the expression at 13 °C overnight. The cells were harvested by centrifugation at 8000 rpm and 4 °C for 15 min, resuspended in 1 mL of ice-cold buffer (50 mM Tris-HCl, pH 8), and disrupted through bead beater. The mixture was centrifuged at 15,000 rpm at 4 °C for 15 min. Crude proteins in cell pellets (insoluble fraction) and supernatant (soluble fraction) were separately resuspended in 200 μL of SDS-PAGE loading buffer (60 mM Tris-HCL pH 6.8, 2% SDS, 0.1% bromophenol blue, 25% glycerine, 14.4 mM β-mercaptoethanol) for electrophoresis [16]. The negative control was *E. coli* SHuffle transformed with pET28a empty vector.

### 2.8. In Vitro Thioesterase Assays

Thioesterases catalyze the release of acyl moieties from the phosphopantetheine of ACP or CoA [30]. As acyl-ACP was hard to obtain, thioesterase assays were done on available acyl-CoA substrates from Sigma-Aldrich (St. Louis, MO, USA) and Avanti Polar Lipids, Inc. (Alabaster, AL, USA). The assay was based on the reduction of 5,5′-dithiobis(2-nitrobenzoic acid) (DTNB, no color) to 5-thio-2-nitrobenzoic acid (TNB, yellow color) [31] by CoASH released from acyl-CoA thioesters [32]. The assay mixture (200 μL) contained 30 mM HEPES, pH 7.5, 1 mM EDTA, 0.3 mM DTNB, 100 μM acyl-CoA, and 300 μg of total crude proteins from the soluble fraction. The blank reference for the assay was the same except for missing the proteins. The negative control was the assay using 300 μg of total crude proteins from the soluble fraction of the strain with empty pET28a as the enzyme source. All the assays took place at 35 °C for 30 min. The absorbance at 412 nm in the reaction was measured by NanoDrop™ 2000/2000c Spectrophotometer. Thioesterase activity was calculated on the absorbance [33]. All assays were done in triplicate.

### 2.9. Site-Directed Mutagenesis

Overlapping PCR was employed for the site-directed mutagenesis of two residues, S96 and H220, separately, in the AT-like domain. The mutagenesis involved two sequential PCR reactions using two flanking primers (AT-like F and R) and two internal primers (AT-like S96A F and R, AT-like H220A F and R) to introduce the mutation (Table S3). The internal primers were used for the first PCR to generate two overlapping mutated nucleotide sequences, which were then used as templates for the second PCR to generate the mutated version of a gene product [34,35]. The mutant AT-like fragments were cloned, independently, into the expression vector pET28a, which were then chemically transformed into *E. coli* SHuffle and *E. coli ∆fadD* strains for enzymatic and free fatty acid analysis.

### 2.10. Statistical Analysis

The statistical analysis of the data from three independent biological replicates were performed using a one-way ANOVA (*p* < 0.05) with multiple comparisons. Additionally, an unpaired *t*-test at *p* < 0.05 was also used to compare activities between SHuffle control and SHuffle AT-like expression on each substrate.

## 3. Results

### 3.1. Sequence Analysis of a AT-Like Domain of the PUFA Synthase

**Figure 1:**
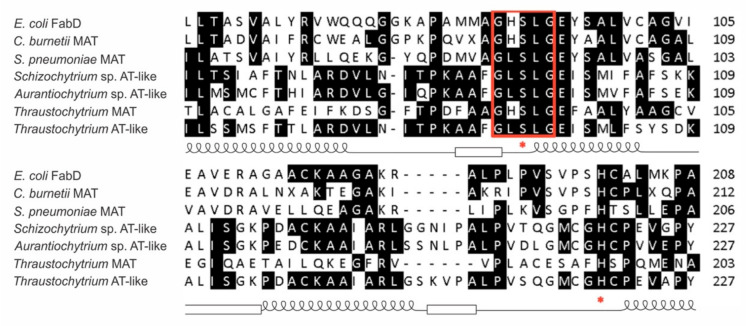
Partial sequence alignment of the acyltransferase (AT)-like domain of the polyunsaturated fatty acids (PUFA) synthase from Thraustochytrium with related sequences from other microorganisms. The AT-like domain has 343 amino acids in length located from amino acid 1048 to 1390 in subunit-B of the PUFA synthase. Here showed only the highly conserved region of AT-like sequence from amino acid 70 to 109 and from 188 to 227 of the 343 amino acids. The predicted α helices (coil) and β strands (square) of the partial α/β hydrolase fold were marked along the sequence. PUFA synthases from *Schizochytrium* (accession no. AAK72880.2, UniProt no. Q94FB7-1) and *Aurantiochytrium* (accession no. AIJ293323.1, UniProt no. A0A076NB29) as well as discrete malonyl-CoA:ACP acyltransferase (MATs) from *Escherichia coli* (accession no. 1mla, UniProt no. P0AAI9), *Coxiella burnetii* (accession no. 3tqe, UniProt no. Q83E39), and *Streptococcus pneumoniae* (accession no. 3im8, UniProt no. Q8DR16) were used in the alignment. The motif GXSXG is highlighted and the conserved serine and histidine residues are depicted by red asterisk.

PUFA synthase for the biosynthesis of DHA in *Thraustochytrium* sp. 26185 comprises three subunits, each with multiple domains [14]. A MAT domain in Subunit-A was highly conserved, while a AT-like domain found in Subunit-B of the PUFA synthase was less conserved in terms of sequence similarity and domain organization among PUFA synthases. For the functional analysis of this AT-like domain, homology search, homology modelling, and multiple sequence alignment were first employed to delimit the domain in the PUFA synthase. The results show that the region of the AT-like domain was located between amino acids 1048 and 1390 in Subunit-B of the PUFA synthase with sequence similarity to a group of hydrolases and transferases including discrete MATs for Type II fatty acid synthesis in bacteria and MAT domains of PUFA synthases in cold/pressure-adapted marine bacteria [36]. All these sequences possessed an α/β hydrolase fold with a conserved motif GXSXG at the putative catalytic site located in a nucleophilic elbow typical for serine hydrolases and transferases. As shown in Figure 1, this motif appeared as GHSLG in most discrete MAT and MAT domains of PUFA synthases, while it appeared as GLSLG in all AT-like domains of PUFA synthases, a typical pentapeptide motif for esterases [37]. Two highly conserved residues, serine at position 96 and histidine at position 220, probably important for activity, were observed in the structure. The two residues were identified as parts of the catalytic triad (Ser-Asp-His) of serine hydrolases in the catalytic sub-domain of the AT-like domain (Figure 1). The domain shares high amino acid identity to the AT-like domains of PUFA synthases from *Schizochytrium* sp. [9] and *Aurantiochytrium* sp. L-BL10 (76.4% and 65.8%, respectively). However, it shares low amino acid identity to discrete MAT proteins from *E. coli* [38], *Coxiella burnetti* [39], and *Streptococcus pneumoniae* [40] (18.9%, 16.2%, and 15.0%, respectively), as well as the MAT domain in Subunit-A of the same PUFA synthase (14.6%). In addition, it also possesses low sequence identity to a group of discrete thioesterases (TE) such as acyl-ACP thioesterases from *Amycolaptosis mediterranei* [41] (16.8%) and *Streptomyces coelicolor* [42] (16.4%).

### 3.2. Expression of the AT-Like Domain in an E. coli fabD Mutant

**Figure 2:**
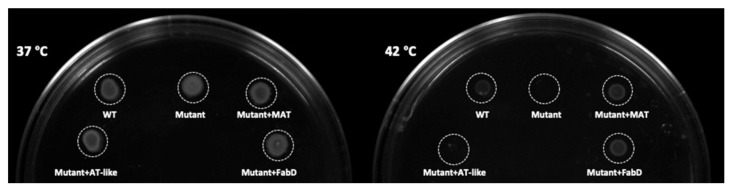
Plate complementation test of the AT-like domain in an *E. coli fabD* mutant grown at permissive (37 °C) and non-permissive (42 °C) temperatures. WT: parental wild type with the empty vector; Mutant: the mutant with the empty vector; Mutant+MAT: the mutant with authentic MAT domain from Subunit-A; Mutant+AT-like: the mutant with the AT-like domain from Subunit-B; Mutant+FabD: the mutant with *E. coli* wild-type MAT gene (*FabD*).

To determine the function of the AT-like domain, this domain from amino acid 1048 to amino acid 1390 in the subunit B of the PUFA synthase was dissected and expressed as a standalone protein in an *E. coli fabD* mutant defective in malonyl-CoA:ACP transacylase (MAT) activity. As MAT is essential for fatty acid synthesis; only a temperature-sensitive mutant of this gene is available in *E. coli* for the complementation test. The wild-type strain (WT) with an empty vector could grow at both permissive (37 °C) and non-permissive temperatures (42 °C), while the mutant with the AT-like domain from Subunit-B (Mutant+AT-like), similar to the mutant with the empty vector (Mutant), could grow at 37 °C, but not at 42 °C. On the other hand, the mutant with either discrete *E. coli MAT* gene *FabD* (Mutant+FabD) or the authentic MAT domain from Subunit-A (Mutant+MAT) could effectively grow at both 37 °C and 42 °C (Figure 2). This result indicates that the AT-like domain is incapable of complementing the defective MAT growth phenotype; thus, it does not function as a malonyl-CoA:ACP transacylase.

### 3.3. Expression of the AT-Like Domain in an E. coli tesAtesB Mutant

As the AT-like domain did not function as malonyl-CoA:ACP transacylase, next we attempted the complementation test of this domain in *E. coli* thioesterase mutants. *E. coli* possesses two major fatty acyl-thioesterases (TesA and TesB), but neither is essential. No changes on growth phenotype and fatty acid profile could be detected in a double mutant (*∆tesAtesB)* [43]. To interrogate whether the AT-like domain could impose any changes on lipid class and fatty acid profile, the domain was expressed as a standalone protein in the double mutant. Fatty acid analysis showed that no significant difference in total fatty acid compositions could be observed among three tested strains: parental wild type, mutant with the empty vector, and mutant with the AT-like domain (Figure S1). Thin layer chromatography analysis showed that no observable difference could be detected on either the type or amount of lipid classes among these strains (data not shown).

### 3.4. Expression of the AT-Like Domain in an E. coli fadD Mutant

To investigate if the AT-like domain has any impact on the production of free fatty acids, another *E. coli* mutant (*fadD*) defective in acyl-CoA synthetase activity was exploited to express the domain (Figure S3). The rationale behind was that the AT-like domain, if functioning as a thioesterase, would increase the accumulation of free fatty acids (FFA) in the mutant where the defect in acyl-CoA synthetase would prevent fatty acid degradation through β-oxidation. As shown in Table 1, expression of the AT-like domain in the mutant increased the production of total free fatty acids by approximately 50% in cell pellets (inside the cells) and more than 30% in supernatants (outside the cells) relative to the mutant control. Among all the free fatty acids produced, the main increase was observed on 11-cis-octadecenoic acid (18:1n-7), a major monounsaturated long chain fatty acid *in E. coli*. The mutant expressing the AT-like domain produced 2 to 3 times 18:1n-7 than the mutant with the empty vector both inside and outside the cells (Figure S2). These results indicate that the AT-like domain might function as a thioesterase by increasing free fatty acid production on the background of defective acyl-CoA synthetase.

### 3.5. In Vitro Activity Assays of the AT-Like Domain

**Figure 3:**
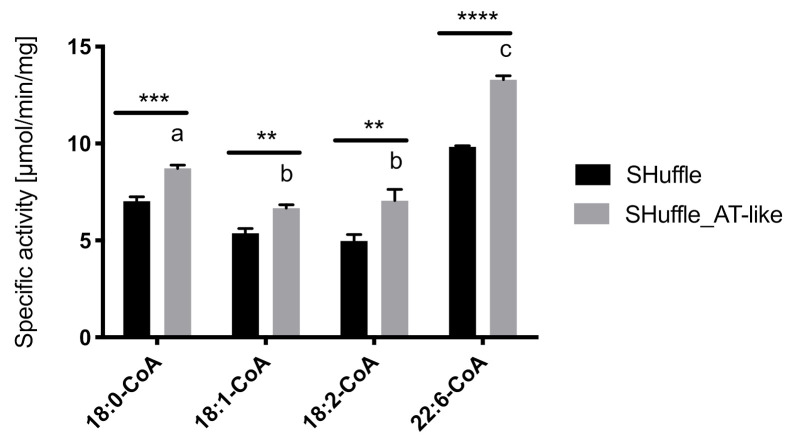
Thioesterase activity of AT-like domain toward four acyl-CoA substrates. Values are reported as means ± standard deviations for three biological replicates. Means with different letters are statistically different according to a one-way ANOVA test (*p* < 0.05) with multiple comparisons. Means between SHuffle and SHuffle AT-like activities are statistically different according to an unpaired *t*-test at *p* < 0.05 (**), *p* < 0.01 (***), and *p* < 0.001 (****).

To confirm if the AT-like domain functions as a thioesterase, this domain was expressed as a standalone protein in an *E. coli* SHuffle strain with enhanced capacity for the appropriate folding and production of expressed proteins. In vitro thioesterase activity assays using the crude protein extract from the strain expressing the AT-like domain as an enzyme source showed that the AT-like domain could hydrolyze all four acyl-CoA substrates tested, stearoyl-CoA (18:0-CoA), octadecenoyl-CoA (18:1-CoA), octadecadienoyl-CoA (18:2-CoA), and docosahexaenoyl-CoA (DHA-CoA). The highest activity was found towards DHA-CoA, followed by 18:2-CoA, 18:0-CoA and 18:1-CoA after normalization against the control (Figure 3). This result indicates that the AT-like domain indeed possesses fatty acyl thioesterase activity.

### 3.6. Mutagenesis Analysis of the AT-Like Domain

Two conserved residues, S96 and H220, were presumably parts of the catalytic triad in the AT-like domain. To investigate the importance of the two residues in catalysis, they were individually replaced by alanine through site-directed mutagenesis. In vitro assays showed that the individual substitutions of the two residues resulted in the reduction of their thioesterase activities towards DHA-CoA, the most preferred substrate tested, by about 80%, relative to the wild-type domain protein (Table 2). Furthermore, expression of the two mutant domains in the *E. coli ∆fadD* strain (Figure S3) also resulted in significant reduction of total free fatty acids relative to those of the controls expressing the empty vector and wild-type domain (Table 3). The extent of reduction of free fatty acids in the cells was more obvious in S96A than H220A. These results indicate these two residues indeed play important roles in catalytic activity.

## 4. Discussion

PUFA synthases for the biosynthesis of VLCPUFAs have been identified in many marine microorganisms such as *Shewanella* sp., *Photobacterium* sp., *Schizochytrium* sp., and *Thraustochytrium* sp. [44]. In Thraustochytrium, a PUFA synthase comprises three large subunits, each with multiple catalytic domains predicted on the presence of characteristic active site residues [14]. However, the exact boundaries and functions of these domains remain undermined. This study undertook sequence analysis, complementation test, in vitro assays, and heterologous expression to investigate the function of a malonyl-CoA:ACP transacylase (MAT)-like domain in Subunit-B of the PUFA synthase in *Thraustochytrium* sp. 21685.

Sequence analysis using a combination of bioinformatic tools indicates the AT-like domain resides between 1048 and 1390 amino acids in the Subunit-B of the PUFA synthase. A GXSXG motif and a histidine residue are conserved among the AT-like domain and related sequences, such as MAT and thioesterase proteins. The conserved serine at position 96 and the histidine at position 220 might be part of a catalytic triad in the active site for catalysis [45], as the substitutions of these two residues with alanine almost abolished the activity detected by an in vitro assay using the crude extract of the expression in *E. coli* as an enzyme source. The residual activity left in the assay could be due to compensated effects of endogenous thioesterases in the host. In fact, the expression of the two mutated AT-like domains in an *E. coli* mutant (*∆fadD*) defective in acyl-CoA synthetase activity confirms that the mutated AT-like domains are not functional, as the amounts of free fatty acids in the mutant strain expressing the two mutated domains are even lower than those in the strain with the empty vector. The lower production of free fatty acids in the transformants with the mutated AT-like domain could be due to the dominant negative mutation effect [46], as functional endogenous *E. coli* thioesterases and non-functional heterologous mutated AT-like domains could compete for the same substrate, leading to the reduced production of free fatty acids.

To functionally analyze the AT-like domain, it was first expressed as a standalone protein in an *E. coli* temperature sensitive MAT mutant (*∆fabD^ts^*). This mutant has been previously used to examine the function of predicted MAT proteins by complementation tests [47]. The result showed that it could not function as a MAT to correct the defective growth phenotype of the mutant, although the AT-like domain shared the highest amino acid identity to malonyl CoA:ACP transacylase proteins. Next, an *E. coli* double mutant (*ΔtesAtesB*) was employed to express the domain, as it has the second highest sequence similarity to thioesterases. However, expression in the mutant did not produce any detectable changes on growth phenotype and lipid/fatty acid profile, similar to the previous result that no difference in growth and fatty acid profile could be observed between the mutant and wild type strain [43]. Subsequently, the AT-like domain was expressed in another *E. coli* mutant (*ΔfadD*) where acyl-CoA synthetase gene was deleted. This mutant has been widely used for enhancing the production of free fatty acids in bacteria [21,48,49,50], as inactivation of acyl-CoA synthetase blocks the formation of acyl-CoA from free fatty acids, thereby preventing fatty acid degradation through β-oxidation. *E. coli* produces fatty acids principally for the biosynthesis of membrane lipids and normally does not accumulate a high level of free fatty acids [50]. Thus, overproduction of free fatty acids in the mutant expressing the AT-like domain is indicative of thioesterase activity.

In marine Thraustochytrids, VLCPUFAs are solely synthesized by an anaerobic pathway catalyzed by a PUFA synthase [14,51]. After synthesis, VLCPUFAs are released as free fatty acids probably catalyzed by thioesterase activity conferred by a domain inside a PUFA synthase [51]. However, identity of this domain in PUFA synthases has not been determined until very recently [52] where the hydrolytic activity of a AT-like domain of protist PUFA synthase for converting acyl-ACPs to free fatty acids was demonstrated by in vitro assays. In addition, acyltransferase-like enzyme, a component of bacterial PUFA synthases, was shown to be the determinant for the molecular species of VLCPUFAs produced by the PUFA synthases [36]. In this study, complementation test in an *E. coli* fabD mutant was first used to show that the AT-like domain of a PUFA synthase from Thraustochytrium does not function as a MAT. The increased production of free fatty acids in an *E. coli* acyl-CoA synthetase mutant expressing the domain then indicated thioesterase activity by releasing free fatty acids from their thioesters. In vitro enzymatic assays using the domain protein expressed in the *E. coli* SHuffle strain as an enzyme source ultimately confirmed that this domain could hydrolyze fatty acyl-CoA substrates with the highest activity towards a thioester of DHA. To prepare the domain protein for in vitro activity assays, we first attempted the expression in a high expression system BL21 (DE3) or Rosetta (DE3). However, all the expressed proteins in these strains appeared in the insoluble fraction (Figure S4). This disallowed us to purify the protein and assay the activity. Afterwards, we expressed it in an *E. coli* strain Shuffle, which has enhanced capacity for appropriate folding. With this strain, we were able to show some activity by in vitro assays using the crude protein. Although the activity is low, the higher activity value of the transformant relative to the empty vector control would indicate the authentic catalytic mode. Collectively, these results from in vitro assays, complementation test, mutagenesis analysis, and production of free fatty acids in an *E. coli ΔfadD* mutant support the notion that the AT-like domain of the PUFA synthase functions as a thioesterase. This activity is probably involved in catalyzing the last step of the VLCPUFA biosynthesis by releasing freshly synthesized DHA attached to ACP domains of the PUFA synthase into a pool of free fatty acids for subsequent metabolisms in Thraustochytrium.

## Figures and Tables

**Table 1:** Free fatty acid (FFA) content in the *∆fadD* mutant expressing the AT-like domain. Values are reported as means ± standard deviations for three biological replicates. Means with different letters in the same row are statistically different according to an unpaired *t*-test (*p* < 0.05).

Source	FFA Content [mg/L]	
	*∆fadD*	*∆fadD*_AT-Like
Intracellular	3.43 ± 0.22 ^a^	4.98 ± 0.003 ^b^
Extracellular	1.29 ± 0.02 ^a^	1.77 ± 0.13 ^b^

**Table 2:** Thioesterase activity of the mutant AT-like domain towards docosahexaenoyl-CoA (DHA-CoA).

Protein	µmol TNB/min/mg Protein
Shuffle control	10.32 ± 0.24 ^a^
Shuffle_AT-like	13.40 ± 0.08 ^b^
Shuffle_AT-like S96A	11.00 ± 0.17 ^c^
Shuffle_AT-like H220A	11.03 ± 0.14 ^c^

Values are reported as means ± standard deviations for three biological replicates. Means with different letters are statistically different according to one-way ANOVA test (*p* < 0.05) with multiple comparisons.

**Table 3:** Free fatty acid contents in *∆fadD_*AT-likeS96A and *∆fadD*_AT-likeH220A. Values are reported as means ± standard deviations for three biological replicates. Means with different letters are statistically different according to a one-way ANOVA test (*p* < 0.05) with multiple comparisons performed among intracellular and extracellular activities, separately.

Source	FFA Content [mg/L]			
	*∆fadD*	*∆fadD*_AT-Like	*∆fadD*_AT-Like S96A	*∆fadD*_AT-Like H220A
**Intracellular**	3.43 ± 0.22 ^a^	4.98 ± 0.003 ^b^	1.75 ± 0.10 ^c^	2.88 ± 0.18 ^d^
**Extracellular**	1.29 ± 0.02 ^a^	1.77 ± 0.13 ^b^	1.32 ± 0.03 ^a^	1.05 ± 0.05 ^c^

## Data Availability

Data sharing not applicable. No new data were created or analyzed in this study. Data sharing is not applicable to this article.

## Supplementary Materials

The following are available online at https://www.mdpi.com/2076-2607/9/3/626/s1, Figure S1: Total fatty acid profile of *E. coli ΔtesAtesB* with AT-like domain and empty vector and parental wild type, Figure S2: Free fatty acids (a) in cell pellets and (b) supernatant of the culture *∆fadD* mutant with the AT-like domain, Figure S3. Expression of AT-like domain, AT-like domain S96A, and AT-like domain H220A in *E. coli ∆fadD* mutant, Figure S4. Expression of AT-like domain in *E. coli* BL21 (D3), Rosetta and Shuffle strains. **Table S1**: Genotypes of *E. coli* mutant and parental strains, Table S2: Plasmids used for AT-like domain cloning, Table S3: Primers used for AT-like domain amplifications and site-directed mutagenesis.
