# Supplementary material for: Functional Analysis of an Acyltransferase-Like Domain from Polyunsaturated Fatty Acid Synthase in Thraustochytrium
Source: Microorganisms. 2021 Mar 17;9(3):626. doi: 10.3390/microorganisms9030626 (PMC8003026; doi:10.3390/microorganisms9030626)
Supplement: Supplementary file 1 [file microorganisms-09-00626-s001.pdf]

Supplemental materials:

Table S1. Genotypes of *E. coli* mutant and parental strains.

Strains	Genotype
***ΔfabD* parental**	*araC14*, *lacY1*, *tsx-57*, *glnX44*(AS), *gltA5*, *galK2*(Oc), *Rac-0, rfbC1*, *rpsL20*(strR), *xylA5*, *mtl-1*, *lldD1*, *thiE1*, *tfr-5*
ΔfabD	F-, *araC14*, *lacY1*, *tsx-57*, *glnX44*(AS), *gltA5*, *galK2*(Oc), *fabD89*(ts), *rpsL20*(strR), *xylA5*, *mtl-1*, *lldD1*, *thiE1*, *tfr-5*
***ΔtesAtesB* parental**	*K-12 F⁻ λ⁻ ilvG⁻ rfb-50 rph-1*
ΔtesAtesB	*F⁻, ΔtesA::cat ΔtesB, λ⁻, λ⁻ ilvG⁻ rfb-50 rph-1*
ΔfadD	*F– ompT gal dcm lon hsdSB(rB–mB–) λ(DE3 [lacI lacUV5-T7p07 ind1 sam7 nin5]) [malB+]K-12(λS) ΔfadD*
**SHuffle**	*F′ lac, pro, lacIQ / Δ(ara-leu)7697 araD139 fhuA2 lacZ::T7 gene1 Δ(phoA)*<br>*PvuII phoR ahpC* galE (or U) galK λatt::pNEB3-r1-cDsbC (SpecR, lacIq) ΔtrxB rpsL150(StrR) Δgor Δ(malF)3*
**BL21 Star (DE3)**	*fhuA2 [lon] ompT gal [dcm] ΔhsdS*
**Rosetta (DE3)**	*F- ompT hsdSB(rB- mB-) gal dcm (DE3) pRARE (CamR)*

Table S2. Plasmids used for AT-like domain cloning.

Plasmids	Relevant genotype	Resistance
**pCDFDuet-1_ORF-B**	T7 promoter, contained ORF-B of the PUFA synthase from *Thraustochytrium sp.* 26185	Streptomycin
**pBAD**	T7 promoter, inducible expression through glucose, glycerol and arabinose	Ampicillin
**pET28a**	T7 promoter. High recombinant proteins expression	Kanamycin

Table S3. Primers used for AT-like domain amplifications and site-directed mutagenesis.

Name	Sequence (5' → 3')	Annotation
AT-like (ORF-B) F	GAATTCATGTACGGCGAGGGC	EcoRI site (underline)
AT-like (ORF-B) R	AAGCTTCTAGAGCTTGACGATGGT	HindIII site (underline)
MAT (ORF-A) F	GAATTCATGTTCAGCGAGGTC	EcoRI site (underline)
MAT (ORF-A) R	AAGCTTCTACCACTTGTCAAAGCC	HindIII site (underline)
FabD (*E. coli*) F	AGATCTATGACGCAATTTGCA	BglII site (underline)
FabD (*E. coli*) R	GAATTCTTAAAGCTCGAGCGC	EcoRI site (underline)
AT-like S96A F	TTCGGCCTCGCCCTCGGCGAGAT	Mutation site (underline)
AT-like S96A R	ATCTCGCCGAGGGCGAGGCCGAA	Mutation site (underline)
AT-like H220A F	AGGGCATGTGCGGCGCCTGCC	Mutation site (underline)
AT-like H220A R	GGCAGGCGCCGCACATGCCCT	Mutation site (underline)
pET28a F	GGCCACGATGCGTCCGGCGTAGAGG	For colony PCR
pET28a R	CAGTGGTGGTGGTGGTGGTGCTCGAGT	For colony PCR
pBAD F	GGGCTAACAGGAGGAATTAACCATGGGGGG	For colony PCR
pBAD R	TTCACTTCTGAGTTCGGCATGGGGTCAGG	For colony PCR

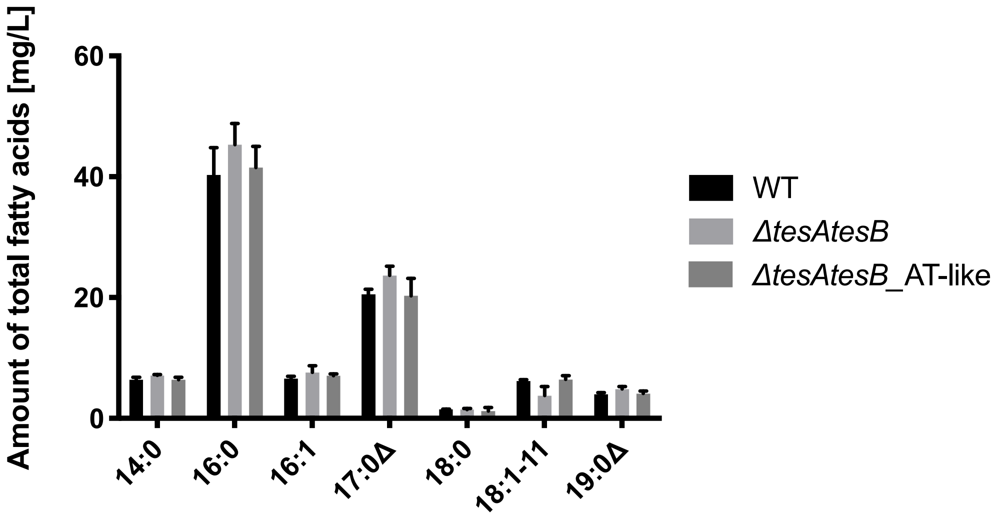

Figure S1. Total fatty acid profile of *E. coli ΔtesAtesB* with AT-like domain and empty vector, and parental wild type. Values are reported as means ± standard deviations for three biological replicates. No statistical significance could be detected in all the fatty acids.

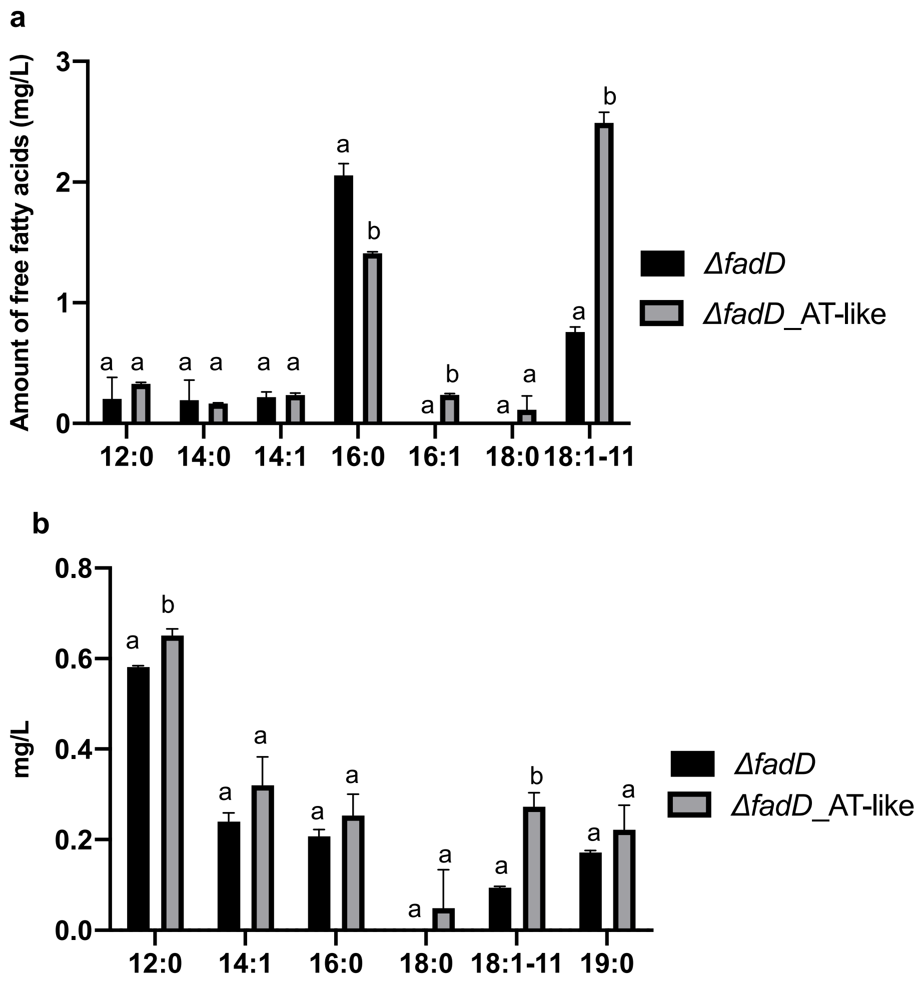

Figure S2. Free fatty acids (a) in cell pellets and (b) supernatant of the culture *ΔfadD* mutant with the AT-like domain. Values are reported as means ± standard deviations for three biological replicates. Means with different letters are statistically different according to an unpaired *t* test ($p<0.05$).

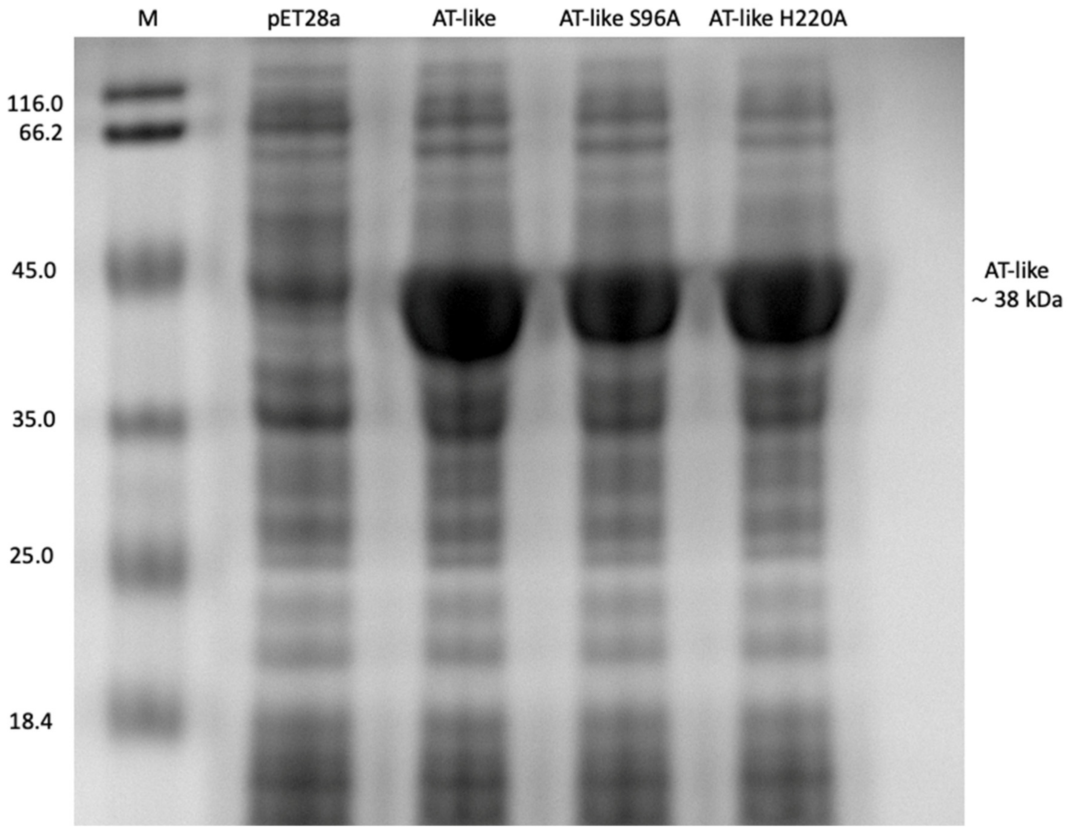

Figure S3. Expression of AT-like domain, AT-like domain S96A and AT-like domain H220A in *E. coli ΔfadD* mutant. M: Marker. pET28a: *E. coli ΔfadD* mutant with empty vector.

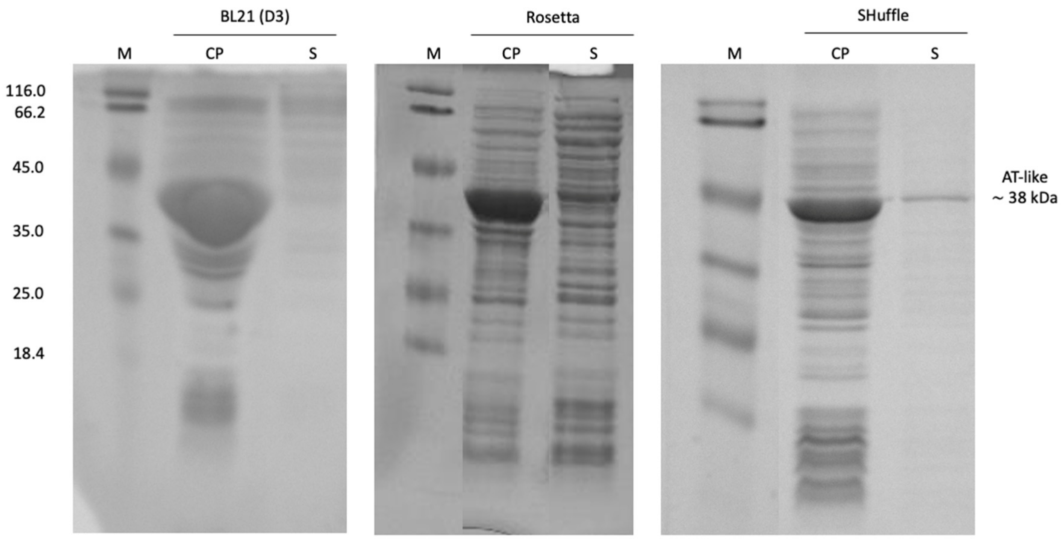

Figure S4. Expression of AT-like domain in *E. coli* BL21 (D3), Rosetta and Shuffle strains. M: Marker. CP: cell pellet (insoluble fraction). S: supernatant (soluble fraction).
